# Trimester two gestational exposure to bisphenol A and adherence to mediterranean diet are associated with adolescent offspring oxidative stress and metabolic syndrome risk in a sex-specific manner

**DOI:** 10.3389/fnut.2022.961082

**Published:** 2022-10-05

**Authors:** Astrid N. Zamora, Elizabeth Marchlewicz, Martha M. Téllez-Rojo, Charles F. Burant, Alejandra Cantoral, Peter X. K. Song, Adriana Mercado, Dana C. Dolinoy, Karen E. Peterson

**Affiliations:** ^1^Department of Nutritional Sciences, University of Michigan School of Public Health, Ann Arbor, MI, United States; ^2^Department of Environmental Health Sciences, University of Michigan School of Public Health, Ann Arbor, MI, United States; ^3^Center for Research on Nutrition and Health, National Institute of Public Health, Cuernavaca, Mexico; ^4^Department of Internal Medicine, Michigan Medicine, Ann Arbor, MI, United States; ^5^Health Department, Iberoamerican University, Mexico City, Mexico; ^6^Department of Biostatistics, University of Michigan School of Public Health, Ann Arbor, MI, United States

**Keywords:** adolescent health, bisphenol A, early-life exposures, mediterranean diet score, metabolic syndrome risk, oxidative stress

## Abstract

**Background:**

Exposure to prenatal bisphenol A (BPA) and Mediterranean Diet Score (MDS) has been linked to metabolic risk in child offspring. It remains unclear if independent and interactive effects persist in adolescence.

**Methods:**

We examined prenatal BPA and MDS on adolescent offspring metabolic syndrome risk score (MRS) and 8-isoprostane (8-iso), a biomarker of oxidative stress. Data from maternal-adolescent dyads from a Mexico City cohort were utilized, including trimester-specific prenatal BPA from spot urine and MDS from food frequency questionnaires. Offspring socio-demographic data and biomarkers to estimate MRS and 8-iso were obtained during peri-adolescence.

**Results:**

Adjusted linear regression models examined associations between trimester-specific BPA, MDS, and BPA^*^MDS on outcomes. Sex-stratified analyses revealed a significant association between MDS with increased 8-iso (β = 0.064, *p* < 0.05), and a marginal association between trimester two BPA with increased 8-iso (β = 0.237), while MDS modified the marginal association between BPA and 8-iso in females (β = 0.046). A negative, marginal association was observed between trimester two BPA and MRS (β = – 0.728), while BPA ^*^ MDS was marginally, positively associated with MRS (β = 0.152) in males.

**Conclusions:**

Study findings indicate that trimester two prenatal BPA and maternal adherence to a Mediterranean diet may have sexually dimorphic effects on adolescent offspring oxidative stress and metabolic syndrome risk.

## Introduction

Prenatal programming of metabolic syndrome (MetS) has been reported in experimental and human epidemiologic studies (1–4). For example, the prenatal maternal environment has been linked to an increased risk of offspring MetS in later life (5, 6). A growing body of mechanistic literature suggests links between perinatal exposure to the endocrine-disrupting chemical (EDC), bisphenol A (BPA), and biological pathways that influence child offspring MetS (7, 8). Ubiquitous in the environment, BPA is found in various sources, including food products from BPA leaching from containers and cans in which foods are stored and packaged (9). Recent human studies have underscored the significant public health implications of these exposures since BPA has been observed in >95% of urine samples of pregnant people in the United States (10, 11). Moreover, *in-utero* BPA exposure has been associated with alterations in early life health outcomes of offspring (12, 13). Additionally, animal studies have found links between perinatal BPA exposure and increased MetS development among adult offspring (14–16). Human and animal study findings provide biological plausibility for examining maternal BPA and offspring MetS.

Human offspring physiological changes with age, such as puberty, pose challenges to assessing MetS among developing adolescents (17). Albeit, oxidative stress has been implicated as an underlying risk factor in the pathogenesis of many chronic metabolic diseases, including MetS (18, 19). For example, obesity is one subsequent condition of systemic oxidative stress in children (20). Moreover, studies suggest that environmental exposures to toxicants, such as phenols, can induce oxidative stress (21). For example, a study of Mexican-American women found links between prenatal exposure to phthalates and increased levels of 8-isoprostane (8-iso) (22). Isoprostanes emerge from free radical-dependent lipid peroxidation and are validated and accepted biomarkers of human oxidative stress (23).

Although prenatal programming research has widely focused on exposures that increase offspring disease risk, such as BPA (24), studies have also documented the protective effects of the maternal environment on offspring health in later life (25). To illustrate, research has shown that maternal adherence to the Mediterranean diet during gestation is protective against markers of metabolic health in offspring (26, 27). Given existing evidence demonstrating offspring metabolic health benefits attributed to adherence to the Mediterranean diet and the harmful impact of prenatal maternal BPA exposure, we must examine whether Mediterranean diet adherence modifies the association between prenatal maternal BPA exposure with MetS risk and oxidative stress in adolescent offspring. Since previous studies have revealed sexually dimorphic metabolic responses to prenatal exposures (28–30), it is plausible that associations between maternal exposure to BPA and Mediterranean diet adherence on offspring metabolic outcomes differ by sex.

To address the lack of human epidemiologic evidence supporting the plausible links between prenatal programming of adolescent offspring MetS by prenatal maternal exposure to EDCs and healthy maternal diet quality, this study utilized a prospective birth cohort to examine the relationship between trimester-specific prenatal maternal exposure to BPA and adherence to a Mediterranean diet on metabolic syndrome risk score (MRS) and oxidative stress among offspring during the period of adolescence. We also sought to evaluate if maternal adherence to a Mediterranean diet modified the association between prenatal maternal BPA exposure with MRS and oxidative stress. We hypothesized that higher exposure to prenatal maternal BPA during pregnancy would be associated with an increased MRS and oxidative stress in female and male offspring and that maternal adherence to a Mediterranean diet would attenuate the association between prenatal BPA exposure with oxidative stress and MRS.

## Materials and methods

### Study population

The analytic study sample included mother-adolescent dyads from two sequential birth cohorts of the Early Life Exposure in Mexico to Environmental Toxicants (ELEMENT) study (31). Between 1997 and 2004, mothers were recruited from hospitals serving low-to-moderate income populations living in Mexico City. Mothers were recruited during their trimester one prenatal visit where maternal height, weight, urinary samples, and questionnaires on food intake and sociodemographic data were collected at each trimester of pregnancy.

**Figure 1:**
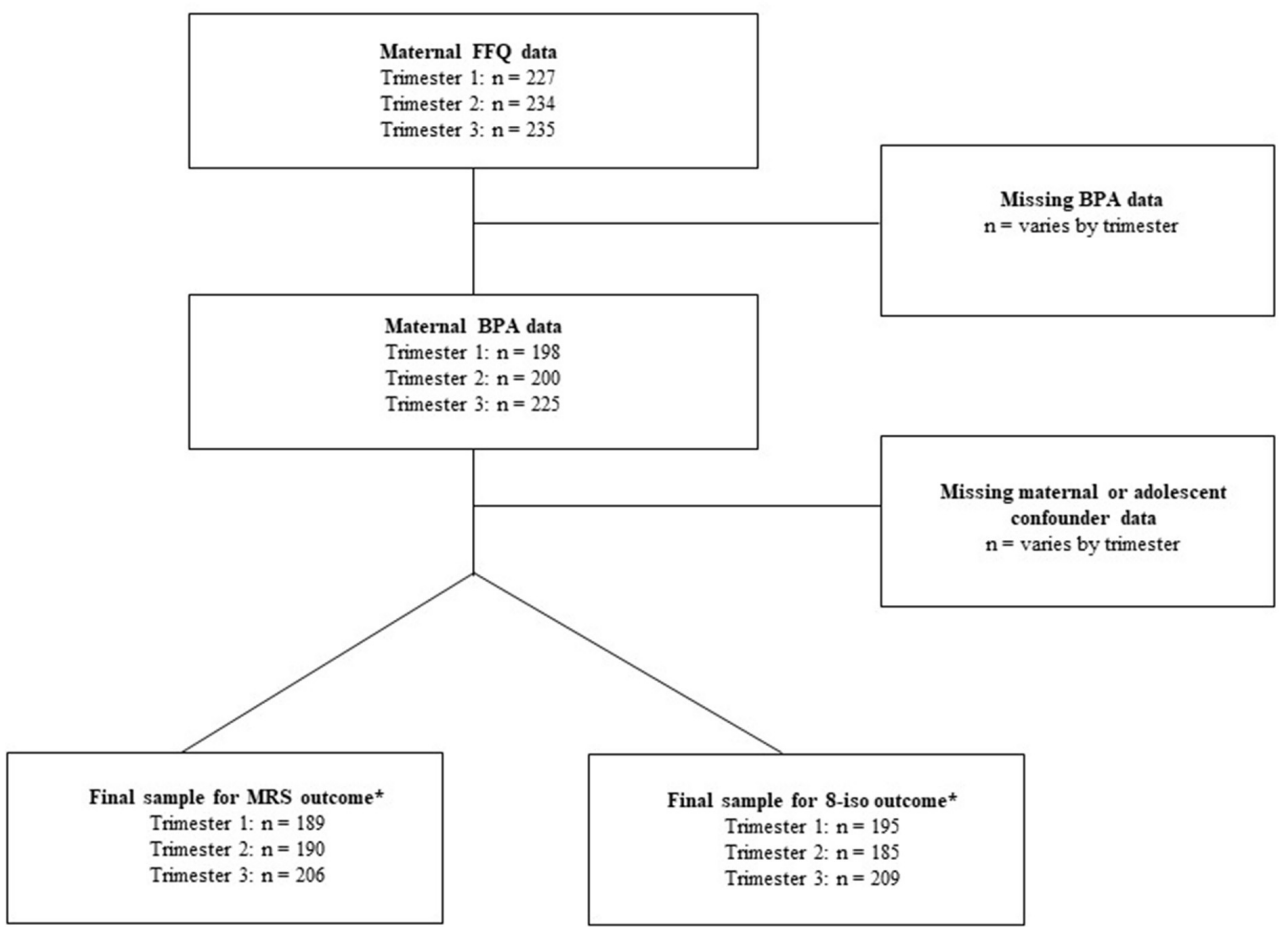
Study flow chart. *Sample sizes from fully adjusted regression models.

Between 2010 and 2012, 250 adolescent offspring participated in a follow-up visit. Measures collected included anthropometry, urine and blood samples for biomarker assessment, food frequency questionnaires, physician-assessed pubertal status, self-reported physical activity, and sociodemographic characteristics. We present a range of sample sizes in the present study depending on data availability. The final analytic sample sizes used for regression analyses varied by trimester based on the availability of exposure, outcome, and confounding variables included in the fully adjusted regression models. Therefore, sample sizes for the MRS outcome at trimesters one, two, and three samples were *N* = 189, *N* = 190, and *N* = 206, respectively. While for the 8-isoprostane outcome, the sample sizes for trimesters one, two, and three were *N* = 195, *N* = 185, and *N* = 209, respectively. See Figure 1 for the study flow chart.

The Mexico National Institute of Public Health Research, Ethics, and Biosafety Committees (approval number 1377) approved all research protocols and procedures, and all participants provided informed consent.

### Exposure biomarker assessment of maternal urinary bisphenol A

Total (free + glucuronidated) BPA was measured from spot urine samples collected from mothers during each trimester of pregnancy using isotope dilution–liquid chromatography-tandem mass spectrometry (ID–LC-MS/MS) at NSF International (Ann Arbor, MI, USA). We chose to measure total BPA to be consistent with gold standards established by the Centers for Disease Control and Prevention (CDC); methods were developed based on CDC protocols as previously described elsewhere (32). During the measurement of prenatal maternal urinary BPA, urinary specific gravity (SG) was also measured using a handheld digital refractometer (ATAGO Company Ltd., Tokyo, Japan) to adjust for variability in urine output and concentration between study participants. SG-corrected prenatal maternal urinary BPA concentrations were calculated for use in specific statistical analyses.

Adolescent urinary BPA concentrations were assessed at NSF International in Ann Arbor, MI, using the same ID-LC-MS/MS method (32) to measure prenatal maternal BPA.

### Mediterranean diet score assessment

During each trimester of pregnancy, mothers completed an interview-administered food frequency questionnaire (FFQ). Intake information was collected by a trained interviewer using a >100 item semi-quantitative FFQ, allowing participants to provide information on typical consumption habits in the past month. The questionnaire used the methodology from Willett (33) and was translated and validated for use among Mexican Spanish-speaking women of reproductive age (33, 34). Participating mothers were required to provide information regarding how often, on average, they consumed each food of standard portion size. Each question had a total of nine response options, including: <1 time/month or never, 1–3 times/month, 1 time/week, 2–4 times/week, 5–6 times/week, 1 time/day, 2–3 times/day, 4–5 times/day, and ≥6 times/day. Raw response frequency of consumption values (1–9) was transformed into semi-continuous variables representing servings per day.

The Trichopoulou Mediterranean diet score (MDS) method was used to assess maternal Mediterranean diet adherence in the present study (35). Prior to coding, we used data from FFQs and compiled similar foods into food group categories used by the MDS method. Mediterranean diet scoring was first proposed by Trichopoulou (36–38). This original MDS uses scoring criteria from 0-9 points, with more points indicating higher adherence to the diet. To compute trimester-specific MDS for each ELEMENT mother in this study, the median daily intake (grams) was calculated for each of the eight food groups: fish, legumes, fruits & nuts, vegetables, whole grains, meat, poultry, and dairy. For “beneficial” foods from the following categories: fish, legumes, fruits & nuts, vegetables, and cereal (assumed to be whole grain), participants with consumption below the median were assigned a value of 0, and those with consumption that was at or above the median were assigned a value of 1. For foods that were considered “detrimental” from the following categories: meat, poultry, and dairy (assumed to be whole fat), participants with consumption that was below the median were assigned a value of 1, while those with consumption at or above the median were assigned a value of 0. Individuals received an additional point if their daily alcohol consumption was within a moderate range (2–25 g/day for women) (36). However, the alcohol consumption category was excluded since alcohol intake was negligible among pregnant women and adolescents in the present study. Thus, the MDS in the present study ranged from 0 to 8 points (Supplementary Table 3). Adolescents completed FFQs, similar to those their mothers completed during pregnancy (39), and MDS was calculated as described above.

### Adolescent metabolic syndrome risk score and oxidative stress

During the 2010–2012 adolescent study visit, fasting glucose, total group triglycerides (TGGs), and high-density lipoprotein cholesterol (HDL-C) were measured using a biochemical analyzer (Cobas Mira Plus, Roche Diagnostics, Basel, Switzerland). Following venous blood collection, serum aliquots were frozen at –80°C and shipped from the Mexico National Institute of Public Health to the Michigan Diabetes Research Center Chemistry Lab for analysis. Serum leptin was measured via RIA (Millipore) and Insulin-like growth factor 1(IGF-1) by chemiluminescence immunoassay (Immulite 1000).

Adolescent anthropometry measured by trained research staff included height, weight, and waist circumference. Seated blood pressure measurements were taken in duplicate, and average systolic blood pressure (SBP) and diastolic blood pressure (DBP) levels were used in the analyses. Fasting venous blood samples were used to estimate serum hormone levels (leptin and IGF-1). Adolescent MetS risk was assessed via MRS previously validated for classifying MetS among adolescents (40). Based on the present sample, sex-specific z-scores were calculated for four adolescent variables: waist circumference, fasting glucose, fasting lipids (TGG/HDL-C), and average blood pressure [(SBP + DBP)/2]. The sex-specific MRS was calculated by averaging the four z-scores.

Serum samples were utilized to quantify 8-iso. Samples were stored at –80°C with 0.005% butylated hydroxytoluene to prevent oxidation during storage, then transferred on dry ice to Cayman Chemical (Ann Arbor, MI, USA). The 8-isoprostane analyses were conducted via a competitive enzyme immunoassay (EIA) kit (No. 516351), with a sensitivity of 2.7 pg/mL and intra-assay coefficient variability of 9.5%. The National Institute of Environmental Health Sciences initiative, Biomarkers of Oxidative Stress Study, determined that F_2_-isoprostanes were readily quantifiable markers of lipid peroxidation that relate to disease-relevant measures (41, 42). Other recent reviews of oxidative stress deemed 8-iso a highly accurate measurement of lipid peroxidation (43, 44).

### Covariates

We considered various maternal and adolescent sociodemographic and behavioral characteristics as potential confounders based on *a priori* knowledge (45–50). Trimester-specific and post-partum body mass index (BMI) were measured, and gestational weight gain was calculated from these measures. At baseline, mothers reported years of education, which served as a proxy for socioeconomic status. We also collected information on maternal age at pregnancy, parity, and delivery type.

Based on *a priori* knowledge demonstrating links between pubertal development with insulin sensitivity (51), leptin (52), and IGF-1 (53), a trained physician measured pubertal status using a composite of physician-assessed Tanner stages recorded for adolescents. Briefly, Tanner staging is a five-point scale of physical pubertal development based on pubic hair (males and females), breast (females), and genital (males) development (54, 55). Puberty was treated as a dichotomous variable, with adolescents classified as peri-pubertal or pubertal (Tanner stage 2 or greater). Because physical activity has also been linked to decreased MetS and insulin resistance risk in adolescents (56), we collected physical activity information from a self-reported questionnaire previously validated in Mexican adolescents (57, 58). The weekly hours spent performing vigorous (e.g., swimming, running, soccer) and non-vigorous (e.g., walking, cleaning) activities were summed, and quartiles were created based on the present sample. Finally, adolescent BPA and MDS were added to predictive models to adjust for current exposures.

### Statistical analyses

To examine differences in the distribution of adolescent MRS according to maternal and adolescent sociodemographic characteristics, we examined differences in the mean and standard deviation (SD) of MRS in the entire sample and stratified by sex. We used the same methodology to investigate differences in the distribution of 8-iso among the entire sample and stratified by offspring sex. We calculated trimester-specific summary statistics (geometric means, SD, and percentiles) to examine the distribution of SG-adjusted prenatal urinary BPA. We also calculated trimester-specific summary statistics to explore the distribution of maternal MDS. Quantile-Quantile plots of residuals were used to assess the normality of all variables and ln-transformed right-skewed variables, including prenatal maternal urinary BPA and 8-iso.

The distribution of prenatal maternal urinary BPA and MDS was explored, and the intraclass correlation coefficients (ICCs) were calculated to compare exposure measures across trimesters. Finally, we provide sex-stratified summary statistics (median and interquartile range) for the distribution of prenatal maternal BPA and MDS, adolescent metabolic outcomes of interest, and study covariates.

Linear regression models were used to assess the relationship between maternal exposures with adolescent offspring MRS and 8-iso. Trimester-specific models were created to examine whether the prenatal timing of environmental exposures altered the metabolic programming of offspring. Separate models at each trimester examined the association between prenatal maternal urinary BPA, MDS, and the interaction between BPA and MDS (i.e., BPA^*^MDS) on adolescent MRS and were compared to other trimesters. The BPA^*^MDS interaction term was included in all models to determine if maternal MDS modified the effect of prenatal BPA exposure on outcomes of interest. We ran unadjusted regression models, only adjusted for specific gravity to explore the relationship between exposure variables and offspring outcomes. Next, we ran linear regression models adjusted for maternal (i.e., trimester specific SG urinary BPA and maternal education) and adolescent characteristics (i.e., pubertal status, current urinary BPA, and MDS). We stratified by sex based on *a priori* knowledge demonstrating the sexually dimorphic effects of BPA.

Statistical significance was determined *a priori* at *P* < 0.05. Due to the small sample size and the exploratory nature of the present analysis, marginal significance was also used to assess the significance of associations and set at *P* < 0.10. All analyses were conducted in SAS 9.4 (Cary, NC, USA).

## Results

The mean (SD) maternal age at pregnancy was 26.8 (5.6) years, and the mean (SD) age for adolescent offspring was 10.3 (1.7) years. Approximately 53% of the adolescent sample was female (Supplementary Table 1).

Differences in the distribution of adolescent MRS according to maternal and adolescent sociodemographic are presented in Table 1. Adolescent MRS differed by maternal weight, with maternal weight gain positively associated with adolescent MRS in females. Adolescent females born to mothers that gained 6.0–8.5 kg had a lower MRS than those born to mothers that gained 8.5–11.0 kg and > 11.0 kg (*p* = 0.03). However, MRS differed by adolescent age, pubertal status, and leptin levels among the entire adolescent sample. Adolescents 8–10 years had a lower MRS than adolescents between 13 and 14 years (– 0.50 vs. 0.30; *p* = 0.03). After sex stratification, the association persisted among male offspring (*p* = 0.04). Similarly, adolescents who had entered puberty had a significantly greater MRS than peri-pubertal adolescents (– 0.12 vs. 0.16; *p* = 0.00). Higher leptin levels were associated with a greater MRS among all adolescents. Adolescents with leptin concentrations <5 ng/mL had a lower MRS compared to those with leptin >15 ng/mL (– 0.51 vs. 0.53; *p* = 0.00). We did not find evidence of differences in adolescent 8-iso levels by maternal or adolescent characteristics (Table 2).

**Table 1:** Differences in the distribution of adolescent metabolic syndrome risk scores (−2 to 2) across maternal and adolescent sociodemographic characteristics.

	**Entire sample (*****N*** = **250)**				**Females (*****N*** = **132)**				**Males (*****N*** = **118)**			
	***N***	**Mean**	**SD**	***P***	***N***	**Mean**	**SD**	***P***	***N***	**Mean**	**SD**	***P***
**Maternal characteristics**												
Age at Pregnancy (years)												
15–24	101	0.005	0.625	0.65	51	0.020	0.594	0.67	50	−0.011	0.661	0.88
25–34	117	−0.026	0.595		60	−0.044	0.546		57	−0.007	0.646	
35–44	30	0.092	0.692		20	0.088	0.764		10	0.099	0.556	
Education (years)												
<10	88	0.035	0.645	0.74	50	0.034	0.611	0.85	38	0.036	0.696	0.62
10–12	125	−0.030	0.596		64	−0.009	0.578		61	−0.051	0.617	
>12	35	0.025	0.638		17	−0.058	0.670		18	0.103	0.616	
Trimester one BMI												
<18	1	−0.525	.	0.58	0	.	.	0.46	1	−0.525	.	0.35
18–24.9	103	−0.052	0.634		48	−0.124	0.537		55	0.012	0.707	
25–29.9	84	0.016	0.632		51	0.099	0.639		33	−0.113	0.608	
30–34.9	25	−0.002	0.551		15	0.047	0.612		10	−0.076	0.464	
>35	8	0.287	0.867		3	−0.066	1.397		5	0.499	0.437	
Gestational weight gain (kg)												
<6	48	0.029	0.639	0.62	28	0.063	0.649	0.03^a^	20	−0.020	0.638	0.17
6–8.5	66	−0.008	0.629		34	−0.195	0.541		32	0.191	0.662	
>8.5–11	55	−0.105	0.549		28	−0.096	0.598		27	−0.114	0.504	
>11	52	0.050	0.714		27	0.264	0.626		25	−0.181	0.743	
**Adolescent characteristics**												
Age (years)												
8–10	172	−0.050	0.651	0.03^a^	93	−0.021	0.661	0.43	79	−0.083	0.641	0.04^a^
11–12	54	0.039	0.528		25	−0.024	0.408		29	0.093	0.616	
13–14	22	0.303	0.464		13	0.208	0.392		9	0.440	0.546	
Pubertal status^1^												
Peri-pubertal	144	−0.116	0.617	0.00^a^	85	−0.115	0.632	0.001^a^	59	−0.117	0.601	0.04^a^
Pubertal	104	0.162	0.584		46	0.215	0.469		58	0.120	0.663	
Leptin (ng/mL)												
<5	62	−0.512	0.411	0.00^a^	18	−0.700	0.329	0.00^a^	44	−0.435	0.419	0.00^a^
5–8	64	−0.135	0.495		30	−0.255	0.388		34	−0.030	0.557	
>8–15	60	0.129	0.519		39	0.014	0.509		21	0.343	0.478	
>15	62	0.529	0.527		44	0.450	0.503		18	0.722	0.546	
Physical activity (METS)^2^												
<17	62	−0.021	0.723	0.83	35	−0.060	0.676	0.58	27	0.030	0.789	0.99
17–26	62	−0.045	0.596		38	−0.060	0.623		24	−0.022	0.563	
>26–40	58	0.054	0.628		27	0.122	0.606		31	−0.005	0.651	
>40	66	0.018	0.525		31	0.039	0.465		35	−0.001	0.579	

1 Sex-specific peri-pubertal (<1) and pubertal (>1) status were determined by physician-assessed Tanner staging for males (genital or pubic hair development) and females (breast or pubic hair development);

2 METs are metabolic equivalent;

a *P* < 0.05, ^b^*P* < 0.10.

**Table 2:** Differences in the distribution of adolescent 8-isoprostane (pg/mL) across maternal and adolescent sociodemographic characteristics.

	**Entire sample (*****N*** = **250)**				**Females (*****N*** = **132)**				**Males (*****N*** = **118)**			
	***N***	**Mean**	**SD**	***P***	***N***	**Mean**	**SD**	***P***	***N***	**Mean**	**SD**	***P***
**Maternal characteristics**												
Age at pregnancy (years)												
15–24	98	523.7	188.4	0.24	48	500.5	151.3	0.79	50	546.1	217.4	0.19
25–34	114	492.4	172.7		59	499.3	167.1		55	484.9	179.7	
35–44	30	468.9	140.8		20	473.8	152.2		10	459.1	121.9	
Education (years)												
<10	85	506.1	198.7	0.51	48	494.4	174.8	0.99	37	521.4	227.5	0.38
10–12	123	508.4	169.7		63	497.7	153.6		60	519.6	185.8	
>12	34	469.8	135.7		16	492.1	128.6		18	450.1	142.3	
Trimester one BMI												
<18	1	444.7	.	0.99	0	.	.	0.94	1	444.7	.	0.98
18–24.9	100	503.6	198.4		47	496.3	159.2		53	510.1	229.0	
25–29.9	81	509.4	170.5		48	506.1	174.8		33	514.3	166.6	
30–34.9	25	489.7	135.0		15	490.3	125.0		10	488.9	155.7	
>35	8	472.5	166.6		3	501.1	200.8		5	455.4	165.6	
Gestational weight gain (kg)												
<6	48	495.4	159.0	0.53	28	486.7	125.5	0.68	20	507.7	199.6	0.18
6–8.5	63	517.1	180.9		32	479.9	157.4		31	555.6	197.6	
>8.5–11	53	524.5	219.2		27	528.8	203.0		26	520.1	239.0	
>11	51	469.4	145.5		26	508.4	154.7		25	428.8	125.8	
**Adolescent characteristics**												
Age (years)												
8–10	168	501.3	181.4	0.31	90	498.6	157.9	0.28	78	504.4	206.2	0.79
11–12	53	523.8	167.9		25	517.1	165.5		28	529.7	172.9	
13–14	21	454.5	149.0		12	429.7	137.3		9	487.5	165.7	
Pubertal status^1^												
Peri-pubertal	141	503.2	191.9	0.91	84	500.3	165.5	0.65	57	507.4	226.7	0.92
Pubertal	100	500.7	152.4		43	486.8	143.7		58	511.1	159.0	
Serum Leptin (ng/mL)												
<5	59	483.7	169.5	0.28	17	475.7	183.2	0.41	42	486.9	165.8	0.64
5–8	64	534.8	200.9		30	524.1	144.3		34	544.3	242.0	
>8–15	60	480.5	166.1		39	466.9	143.7		21	505.6	202.8	
>15	59	507.4	162.1		41	510.7	169.4		18	499.7	148.2	
Physical Activity (METS)^2^												
<17	60	518.8	167.2	0.74	34	548.6	176.4	0.15	26	479.8	148.6	0.73
17–26	60	494.1	198.9		36	482.5	161.4		24	511.6	247.5	
>26–40	57	510.2	181.9		26	476.4	131.4		31	538.7	213.4	
>40	65	487.1	158.3		31	469.3	146.5		34	503.3	168.9	

ABR: BMI, body mass index;

1 Sex-specific peri-pubertal (<1) and pubertal (>1) status were determined by physician-assessed Tanner staging for males (genital or pubic hair development) and females (breast or pubic hair development;

2 METs are metabolic equivalent.

The distribution of maternal and adolescent urinary BPA concentration and MDS are presented in Table 3. We found that the median (50th percentile) prenatal maternal urinary BPA concentrations ranged from 1.1 to 1.5 ng/mL; concentrations did not differ by pregnancy trimester (Table 3). However, the distribution of urinary BPA varied across the trimester, as evident by the large ICCs (Supplementary Table 2). Median maternal MDS did not differ across trimesters (Table 3). Although the ICCs comparing MDS during trimester one vs. trimesters two and three were low (– 0.03 and 0.11, respectively), the higher ICC comparing trimester two and three (0.44, *p* < 0.05) suggests that maternal diet fluctuated more between trimester one and two, and then stabilized during trimester two and three (Supplementary Table 2).

**Table 3:** Distribution of exposure variables from prenatal and adolescent exposure periods.

	**N**	**GM**	**GSD**	**Min**	**25%**	**50%**	**75%**	**Max**
**Urinary BPA (ng/mL), SG-adjusted**								
**Maternal measures**								
Trimester one	198	1.6	2.2	0.2	0.9	1.5	2.8	23.4
Trimester two	200	1.7	2.1	0.2	1.0	1.5	2.5	30.3
Trimester three	225	1.3	2.0	0.3	0.8	1.1	1.8	23.4
**Adolescent measures**	242	1.6	2.2	0.4	0.9	1.4	2.6	27.7
	**N**	**Mean**	**SD**	**Min**	**25%**	**50%**	**75%**	**Max**
**Mediterranean diet score (0–8)**								
**Maternal measures**								
Trimester one	227	4.7	1.5	2.0	4.0	5.0	6.0	8.0
Trimester two	234	4.7	1.5	1.0	4.0	5.0	6.0	8.0
Trimester three	235	4.8	1.6	0.0	4.0	5.0	6.0	8.0
**Adolescent measures**	250	3.8	1.5	0.0	3.0	4.0	5.0	8.0

ABR: BPA, Bisphenol A; SG, Specific gravity; GM, Geometric mean; GSD, Geometric standard deviation; Min: minimum, 25%: 25th percentile; 50%: 50th percentile; 75%: 75th percentile; Max: maximum.

Results from unadjusted and adjusted linear regression analyses between maternal exposures and adolescent offspring MRS among the entire sample and stratified by offspring sex are presented in Table 4. We did not observe any significant associations in the unadjusted models. However, after adjustment for *a priori* covariates and sex-stratification, we found an inverse association between trimester two prenatal urinary BPA and MRS in male offspring. To illustrate, every 1-unit increase in ln-transformed trimester two maternal urinary BPA was marginally associated with a 72.8% (β = – 0.728; *p* < 0.10) decrease in adolescent male MRS. Results also revealed that exposure to MDS modified the association between BPA and MRS, with the interactive effect being marginally associated with a 15.2% (β = 0.152; *p* < 0.10) increase in adolescent male MRS. The patterns between trimester two maternal exposures and MRS in the unstratified sample and the sample of female adolescents were consistent with those observed in male adolescents. However, the results were not statistically significant (Table 4).

**Table 4:** Percent changes in adolescent MRS per 1-unit increase in ln-transformed prenatal maternal urinary BPA.

**Trimester of exposure**	**Adolescent metabolic syndrome risk score**							
	**Model 1: Unadjusted**				**Model 2:** ***A priori*** **covariates**			
	**N**	**BPA**	**MDS**	BPA*MDS	**N**	**BPA**	**MDS**	BPA*MDS
		**% Δ**	**% Δ**	**% Δ**		**% Δ**	**% Δ**	**% Δ**
**Entire sample**								
Trimester one	196	−0.101	0.000	0.009	189	−0.086	0.000	0.006
Trimester two	197	−0.324	−0.013	0.066	190	−0.324	−0.007	0.062
Trimester three	222	−0.139	0.032	0.016	206	−0.138	0.025	0.015
**Females**								
Trimester one	106	−0.186	0.011	0.032	103	−0.215	−0.005	0.043
Trimester two	107	−0.113	0.047	0.012	104	−0.262	0.007	0.041
Trimester three	115	0.278	0.060	−0.058	112	0.391	0.044	−0.080
**Males**								
Trimester one	90	0.174	−0.012	−0.059	86	0.436	0.024	−0.132
Trimester two	90	−0.043	−0.002	0.017	86	−0.728^b^	−0.025	0.152^b^
Trimester three	107	−0.422	0.006	0.070	103	−0.286	0.032	0.040

ABR: MRS: Metabolic Syndrome Risk Score; BPA: Bisphenol A; MDS: Mediterranean diet score Model 1: Unadjusted (only adjusted for trimester specific SG urinary BPA); Model 2: Maternal variables (adjusted for trimester specific SG urinary BPA, education level) and adolescent variables (pubertal status, urinary BPA and Mediterranean diet score adherence);

^a^p < 0.05,

b p < 0.10.

Table 5 presents unadjusted and adjusted regression analyses between maternal exposures and adolescent offspring 8-isoprostane among the entire sample and stratified by offspring sex. We observed several significant and marginal associations in the unadjusted models. However, only a few associations remained significant after adjusting for *a priori* covariates. For example, results revealed that higher exposure to maternal BPA during trimester two was marginally associated with a 20.3% (β = 0.203; *p* < 0.10) increase in adolescent 8-iso among the entire sample. Albeit, after sex-stratification, the association persisted in female adolescents only. To illustrate, every 1-unit increase in trimester two ln-transformed prenatal maternal urinary BPA was marginally associated with a 23.7% (β = 0.237; *p* < 0.10) increase in female 8-isoprostane. Moreover, trimester two prenatal maternal MDS was significantly associated with a 6.4% (β = 0.064; *p* < 0.05) increase in 8-isoprostane. Finally, exposure to trimester two MDS modified the association between BPA and 8-isoprostane, with the interactive effect being marginally associated with a decrease in female offspring 8-isoprostane (β = – 0.046; *p* < 0.10). A similar pattern was observed between trimester two exposures and male offspring 8-isoprostane levels. However, these findings were not statistically significant.

**Table 5:** Percent change in adolescent 8-isoprostane per 1-unit increase in urinary ln-transformed prenatal maternal urinary BPA.

**Trimester of exposure**	**Adolescent 8-isoprostane**							
	**Model 1: Unadjusted**				**Model 2:** ***A priori*** **covariates**			
	**N**	**BPA**	**MDS**	BPA *MDS	**N**	**BPA**	**MDS**	BPA *MDS
		**% Δ**	**% Δ**	**% Δ**		**% Δ**	**% Δ**	**% Δ**
**Entire Sample**								
Trimester one	192	0.162	−0.002	−0.024	185	0.079	−0.019	−0.004
Trimester two	186	0.114	0.011	−0.009	185	0.203^b^	0.009	−0.025
Trimester three	216	0.054	0.010	0.001	209	0.078	−0.001	−0.001
**Females**								
Trimester one	103	−0.040	0.005	0.008	100	−0.089	0.004	0.019
Trimester two	103	0.239^b^	0.066^a^	−0.048^b^	100	0.237^b^	0.064^a^	−0.046^b^
Trimester three	111	0.205	0.034^b^	−0.034	108	0.186	0.024	−0.027
**Males**								
Trimester one	89	0.544^a^	−0.011	−0.092^a^	85	0.381	−0.041	−0.052
Trimester two	89	0.084	−0.027	0.009	85	0.228	−0.056	−0.011
Trimester three	105	−0.089	−0.014	0.038	101	−0.034	−0.033	0.029

ABR: BPA: Bisphenol A; MDS: Mediterranean diet score Model 1: Unadjusted (only adjusted for trimester specific SG urinary BPA); Model 2: Maternal variables (adjusted for trimester specific SG urinary BPA, education level) and adolescent variables (pubertal status, urinary BPA, and Mediterranean diet score adherence);

a p < 0.05,

b p < 0.10.

## Discussion

We examined the association between prenatal maternal urinary BPA and adherence to a Mediterranean diet on adolescent offspring metabolic syndrome risk and 8-isoprostane levels among a prospective birth cohort of Mexico City mother-adolescent dyads. To our knowledge, this is the first human epidemiologic study to examine the interactive effects of maternal EDC-diet exposure on multiple markers of metabolic health among adolescent offspring. Sex-specific findings revealed that higher exposure to trimester two BPA was marginally associated with an increase in 8-iso in female offspring, while trimester two MDS was significantly associated with a decrease in 8-iso in female offspring. However, trimester two MDS modified the association between BPA and 8-iso by decreasing levels in females. Contrary to our hypothesis, higher exposure to prenatal urinary BPA and MDS during trimester two was marginally associated with a decrease in male offspring MRS. However, the interactive effect of exposures was marginally associated with increased MRS in males.

The present study adds to the sparse literature on human prenatal programming research by documenting links between prenatal maternal BPA and MDS on subsequent lipid peroxidation in human adolescent offspring. In line with our hypothesis, results from the present study revealed that prenatal BPA exposure during trimester two was marginally associated with an increase in female adolescent offspring oxidative stress (i.e., 8-iso). Although the mechanism underlying this marginal association remains unclear, the authors posit that the finding may be explained by other factors, including adolescent body weight, which could induce an oxidative stress response. This idea is corroborated by an animal study that found that perinatal BPA exposure in rats led to body weight and insulin resistance in later life, which resulted in increased oxidative stress (59). Moreover, in line with this study finding, a recent animal study reported that prenatal BPA exposure was associated with increased levels of a different marker of oxidative stress (i.e., 3-nitrotyrosine levels) in cord blood samples of human offspring (60). However, the study did not follow offspring after birth to determine if longitudinal associations persisted through adolescence, nor did the researchers report sex-specific findings. Further supporting our sex-stratified finding on the association between BPA and oxidate stress, precedence for perinatal BPA exposure leading to later life oxidative stress has been reported in murine model studies (61). For example, perinatal BPA was correlated with higher oxidative stress markers in puberty (61). Notably, the positive association between BPA and oxidative stress only persisted in female offspring after stratification by sex. This sex-specific finding is consistent with sexually dimorphic effects observed in an animal study, which found that perinatal BPA was linked to oxidative stress in only one sex but not the other (59). The comparison animal study contrarily revealed that exposure was associated with male offspring oxidative stress but not female offspring oxidative stress (59). We posit that differences in sex-specific results between the present study and comparison study may be related to various factors. First, likely due to differences in study design and population, with the comparison study being conducted with mice exposed to BPA in a controlled environment, while the present study was observational and conducted among humans. Another potential reason for sex-specific differences could be when the outcomes were assessed, with the animal study measuring oxidative stress during adulthood. In contrast, the present study examined outcomes when adolescents were in peri-puberty. A study among adolescent girls revealed that aside from external exposures, higher BMI increased oxidative stress biomarkers over the pubertal windows for girls (62). In addition, experimental research suggests that sex-differences exist in oxidative stress (63), with research underlying mechanisms that explain sex-differences noting that sex-differences in oxidative stress depend on females having higher antioxidant defense and males having increased reactive oxygen species (ROS) generation (63).

Our study also found that trimester two MDS was significantly associated with increased levels of 8-iso in female offspring, contrary to our hypotheses and most existing research. Although it is unclear why a higher Mediterranean Diet Score (i.e., a healthy diet) was significantly associated with increased oxidative stress in female offspring, the authors posit that these findings could be due to unmeasured confounding. For example, one confounder could have been excessive caloric intake during gestation, which has been associated with a substrate-induced increase in citric acid cycle activity, which results in excess of mitochondrial nicotinamide adenine dinucleotide (NADH) and ROS (64), resulting in oxidative stress of both the mother and offspring. Contrary to our study finding, a recent systemic review revealed that higher adherence to the Mediterranean diet consistently protected offspring against cardiometabolic risk, such that improved adherence to the Mediterranean diet during pregnancy showed lower systolic and diastolic blood pressures in offspring (25). In the present study, we also found that exposure to MDS modified the association between BPA and oxidative stress in females. The interactive effect of BPA and MDS during trimester two was marginally protective against oxidative stress in females. This finding is difficult to compare to other studies, given the lack of existing human studies. However, our recent mouse study demonstrated that maternal exposure to BPA and the Mediterranean diet was associated with higher levels of female offspring TGGs at postnatal day 10 (30). However, the interaction of these components was associated with a decrease in TGGs, though the results were not statistically significant. Overall, our findings revealed that adherence to a healthy, Mediterranean diet may have resulted in oxidative stress due to unmeasured confounding, including excessive caloric intake during gestation, which can be harmful even if the diet pattern being consumed is considered healthy. In addition, our marginal findings indicate that exposure to a healthy, Mediterranean diet during gestation may play a role in modifying the harmful effects of BPA exposure and subsequent female adolescent oxidative stress.

Contrary to our hypothesis that prenatal BPA would be associated with increased adolescent MRS, our study findings revealed that prenatal urinary BPA was associated with decreased MRS in males. A substantial body of literature has revealed that higher exposure to BPA can negatively affect human health (65, 66). However, other studies have also revealed that the effect of prenatal BPA exposure on metabolic outcomes is often monotonic, with different doses of exposure having various influences on metabolic parameters (67). Our finding, which did not align with most existing literature, could be partially explained by several factors, including the fact that we estimated MRS using an average of four metabolic parameters: waist circumference, fasting glucose, lipids, and blood pressure measures. This is important to consider, given that studies have found negative associations between maternal exposure to other EDCs, such as phthalates and child offspring blood pressure (68), as well as *in-utero* BPA exposure and lower offspring weight (69, 70); thus, one or more of the components that were used to estimate MRS could have biased the measurement in one direction. Future studies should consider looking at each metabolic component used to calculate metabolic risk to determine whether association variation exists. This study was among the first to examine this research question and its observational nature, so specific recommendations for pregnant women are not yet clear. However, an existing body of literature from animal and human studies indicates that maternal exposure to BPA can have detrimental effects on the mother, fetus, and offspring in later life (71–74). Therefore, our unexpected study finding does not support the idea that gestational exposure to BPA is beneficial. The authors would not recommend that pregnant women increase their exposure to BPA.

In addition, MDS was not associated with male offspring MRS. However, results revealed that MDS marginally modified the association between BPA and MRS, with the interactive effect of BPA and MDS revealing a marginally positive association with male adolescent MRS. Surprisingly, results revealed that a healthier gestational diet was positively associated with offspring MRS, contrary to existing literature consistently showing that a healthier prenatal diet can have protective effects on offspring cardio-metabolic health (25). However, as previously mentioned, the authors posit that excessive caloric intake of the Mediterranean diet could have negatively impacted metabolic development, which followed offspring during later life. Albeit caloric intake was not accounted for in the present study. In contrast to our current finding, our recently published mouse study (30) found that the *in-utero* interactive impact of exposure to a Mediterranean diet and BPA was not protective against poor metabolic health in 10-month-old male offspring mice (30). Comparing these studies should be done with caution given differences in study design, population and timing of exposures and outcomes.

In terms of comparing our findings to other human studies, one human epidemiologic study among a Turkish cohort found a link between higher BPA and metabolic syndrome in pre-pubertal obese children (75). However, in the study, researchers did not conduct sex-stratified analyses to determine if there were differences between male and female children. Conflicting findings between animal and human studies suggest that the timing and life stage of exposure to BPA may be critical for determining health impact (76, 77). Despite our original hypothesis that prenatal BPA would be associated with increased adolescent MRS, a recent study investigating the impact of prenatal and concurrent BPA exposure on adolescent lipid profile (total cholesterol, TGGs, HDL-C) among the present cohort reported that BPA did not affect lipid profiles (55). Additionally, another study among the present cohort reported that trimester three BPA exposure was not correlated to BMI z-score and skinfold thickness measures (78). These variable findings among multiple birth cohorts warrant additional investigation into the potential effects of prenatal BPA exposure on offspring metabolic risk in late adolescence.

The present study had many strengths. First, the longitudinal, observational nature allowed for repeated maternal urinary BPA exposure and dietary measures. We compared exposure and outcome associations across all three trimesters of pregnancy by obtaining repeated exposures. The trimester-specific measures evaluated critical windows of susceptibility to each exposure; this proved insightful since trimester two maternal BPA exerted a more significant effect, while trimesters one and three were not predictive of adolescent health outcomes. Another strength included using urinary BPA as a biomarker of exposure rather than relying on self-reported BPA exposure. This study also used multiple objective metabolic markers to assess adolescent offspring MRS and oxidative stress.

Limitations of this study included the observational nature; thus, causality could not be assumed. Another limitation was that we collected one spot urine sample during each trimester to estimate maternal exposure to BPA. Urinary BPA concentrations vary by recent exposure; thus, a single urine sample may not represent actual BPA concentrations. However, averaging BPA measures across the three trimesters of pregnancy may have reduced this potential inaccuracy. Similarly, we controlled for prenatal specific gravity (SG) to adjust for urinary dilution. However, studies have shed light on the limitations of adjusting for specific gravity, such as specific gravity varying by BMI. However, there are many strengths to adjusting for specific gravity among pregnant women (79). To illustrate these strengths, in comparison to creatinine, specific gravity is a more appropriate correction approach for studies of pregnant women because of better within-person reproducibility and less affected by participant demographics (79). Another limitation was that health outcomes measured at a single time point during adolescence could not provide insight into possible alterations in metabolic health over time. In particular, puberty and transition to young adulthood is an extended developmental period over which several different metabolic risk factors may emerge (80). Thus, comparing offspring metabolic health measures across the pubertal transition would be more instructive for determining if prenatal exposures impact offspring health at specific times or if the trajectory of MRS and 8-isoprostane changes over time based on prenatal BPA exposure. Another potential limitation of our study was the use of the MDS, which was not developed to accommodate the eating habits of the Mexican population. Given that eating habits are strongly influenced by culture (81), such as food consumption norms, modes of food preparation that are specific to a culture, and other culturally driven factors that influence how a population eats, it is possible that using MDS did not capture the gestational dietary patterns of mothers enrolled in the present study. Moreover, phenols, including BPA and other endocrine-disrupting chemicals (EDCs), such as phthalates, are widespread in the environment (82). The exposure route to these chemicals is likely to occur via dietary intake, with phenol residuals found in fruit and meat products (83). Whether the diet is the primary source of exposure to these chemicals remains unclear (84). Nonetheless, sufficient evidence indicates that chemical mixture exposure is more likely to occur than exposure to single chemicals in isolation (85). Thus, one potential limitation of this study was that we solely examined exposure to BPA and its interaction with MDS. However, participating mothers were likely exposed to mixtures of EDCs, which may have masked the actual effect of BPA alone. Future studies among the present cohort will consider mixtures of EDCs in relation to metabolic health of mother-adolescent dyads from the ELEMENT cohort, including the potential for diet to modify these associations. Finally, given strong evidence that the prenatal maternal environmental is associated with increased risk of offspring MetS in later life (5, 6), including adolescence, future studies should examine how adolescent MRS correlates with adult risk for metabolic syndrome.

In conclusion, our study findings suggest that trimester two exposure to prenatal BPA and a healthy diet have a sexually dimorphic impact on adolescent offspring oxidative stress (females) and MetS risk (males). Understanding the impact of the *in-utero* environment on adolescent offspring metabolic health is critical to informing future interventions that aim to prevent MetS risk and oxidative stress in offspring. Future studies among diverse cohorts of mother-adolescent dyads should utilize a longitudinal framework with multiple oxidative and metabolic health biomarkers to determine if associations persist.

## Data availability statement

The datasets generated for this study will be made available upon reasonable request to the senior author (KP: karenep@umich.edu).

## Ethics statement

The studies involving human participants were reviewed and approved by Mexico National Institute of Public Health Research, Ethics, and Biosafety Committees (approval number 1377). Written informed consent to participate in this study was provided by the participants' legal guardian/next of kin.

## Author contributions

Conceptualization: AZ, EM, DD, and KP. Methodology: EM, MT-R, CB, DD, and KP. Formal analysis: EM and PS. Investigation: EM, AC, and AM. Resources and project administration: MT-R, AM, DD, and KP. Data curation: PS. Writing—original draft preparation: AZ and EM. Writing: AZ, EM, MT-R, CB, AC, PS, AM, DD, and KP. Visualization: AZ. Supervision: DD and KP. Funding acquisition: MT-R, DD, and KP. All authors contributed to the article and approved the submitted version.

## Funding

This research was funded by National Institute of Environmental Health Sciences, grant numbers, R01ES007821, P01ES022844, P30ES017885, R24ES028502, R24ES028502 Supplement, and R35 ES031686]; the U.S. Environmental Protection Agency, grant R835436; Consejo Nacional De Ciencia Y Tecnología [grant 4150M9405]; and by the Consejo de Estudios para La Restauracion y Valoracion Ambiental, Department of Federal District, Mexico. This research was also supported and partially funded by the National Institute of Public Health/Ministry of Health of Mexico. The contents of this publication are solely the grantee's responsibility and do not necessarily represent the official views of the US Environmental Protection Agency or the National Institute of Environmental Health Sciences. Further, the US Environmental Protection Agency does not endorse purchasing any commercial products or services mentioned in the publication.

## Conflict of interest

The authors declare that the research was conducted in the absence of any commercial or financial relationships that could be construed as a potential conflict of interest.

## Publisher's note

All claims expressed in this article are solely those of the authors and do not necessarily represent those of their affiliated organizations, or those of the publisher, the editors and the reviewers. Any product that may be evaluated in this article, or claim that may be made by its manufacturer, is not guaranteed or endorsed by the publisher.
