# Supplementary material for: Trimester two gestational exposure to bisphenol A and adherence to mediterranean diet are associated with adolescent offspring oxidative stress and metabolic syndrome risk in a sex-specific manner
Source: Front Nutr. 2022 Oct 5;9:961082. doi: 10.3389/fnut.2022.961082 (PMC9579372; doi:10.3389/fnut.2022.961082)
Supplement: Supplementary file 1 [file Data_Sheet_1.docx]

Supplementary Material

# Supplementary Data

**Supplementary Table 1.** Study population characteristics

|  | Mean (SD) or % |
| --- | --- |
| **Maternal Characteristics**  Age (years) | 26.8 (5.6) |
| **Adolescent Characteristics**  Age (years) | 10.3 (1.7) |
| Leptin (ng/mL) | 11.3 (9.0) |
| IGF-1 (ng/mL) | 257.1 (104.5) |
| Sex |  |
| *Female* | 52.8 |
| *Male* | 47.2 |
| Pubertal Status |  |
| *Peri-pubertal Female* | 65.2 |
| *Peri-pubertal Male* | 50.8 |
| *Pubertal Female* | 34.8 |
| *Pubertal Male* | 49.2 |

ABR:SD: Standard deviation; IGF-1: Insulin-like Growth Factor-1

**Supplementary Table 2.** Comparison of prenatal maternal BPA and MDS across pregnancy trimesters

| Intraclass Correlation Coefficient (ICC) between pregnancy trimesters | | | | | | |
| --- | --- | --- | --- | --- | --- | --- |
|  | Trimester one | | Trimester two | | Trimester three | |
|  | Coefficient | (95% CI) | Coefficient | (95% CI) | Coefficient | (95% CI) |
| SG-adjusted BPA (ng/mL) | 0.2 | (0.1 - 0.4) | 0.3 | (0.2 - 0.4) | 0.1 | (-0.0 - 0.3) |
| MDS | 0.1 | (-0.0 - 0.2) | 0.4 | (0.3 - 0.5) | -0.0 | (-0.2 - 0.1) |

ABR: BPA: Bisphenol A; SG: Specific gravity; MDS: Mediterranean diet score

**Supplementary Table 3.** Mediterranean diet score calculations

| Food Group | Median Daily Intake (grams) | | | Scoring | |
| --- | --- | --- | --- | --- | --- |
|  | Trimester one | Trimester two | Trimester three | > Median | < Median |
| Beneficial Foods | | | | | |
| Fish | 44.4 | 44.4 | 41.0 | 1 | 0 |
| Legumes | 131.5 | 135.1 | 135.1 | 1 | 0 |
| Fruits & Nuts | 1214.8 | 1350.2 | 1074.3 | 1 | 0 |
| Vegetables | 799.7 | 722.4 | 667.9 | 1 | 0 |
| Whole Grains | 12.95 | 15.3 | 13.0 | 1 | 0 |
| Detrimental Foods | | | | | |
| Meat | 218.4 | 206.0 | 204.5 | 0 | 1 |
| Poultry | 78.6 | 78.6 | 78.6 | 0 | 1 |
| Dairy | 1102.7 | 1280.7 | 1268.0 | 0 | 1 |
